# Comparative Effectiveness Associated With Buprenorphine and Naltrexone in Opioid Use Disorder and Cooccurring Polysubstance Use

**DOI:** 10.1001/jamanetworkopen.2022.11363

**Published:** 2022-05-10

**Authors:** Kevin Y. Xu, Carrie M. Mintz, Ned Presnall, Laura J. Bierut, Richard A. Grucza

**Affiliations:** 1 Health and Behavior Research Center, Department of Psychiatry, Washington University School of Medicine, St. Louis, Missouri; 2 Alvin J. Siteman Cancer Center, Barnes Jewish Hospital and Washington University School of Medicine, St. Louis, Missouri; 3 Departments of Family and Community Medicine and Health and Outcomes Research, St. Louis University, St. Louis, Missouri

## Abstract

**Importance:**

Despite prevalent polysubstance use, treatment patterns and outcomes for individuals with opioid use disorder (OUD) and cooccurring substance use disorders (SUD) are understudied.

**Objective:**

To evaluate the distribution of buprenorphine and naltrexone initiation among individuals with OUD with vs without cooccurring SUD and to assess the comparative effectiveness associated with buprenorphine and naltrexone against drug-related poisonings.

**Design, Setting, and Participants:**

This observational comparative effectiveness study used insurance claims from 2011 to 2016 from the US IBM MarketScan databases to study initiation of medications for OUD (MOUD) among treatment-seeking individuals aged 12 to 64 years with a primary diagnosis of OUD. Cooccurring SUD was defined as SUD diagnosed concurrent with or in the 6 months prior to OUD treatment initiation. Treatment was codified as psychosocial treatment without MOUD or initiation or buprenorphine or naltrexone (including extended-release or oral). Methadone recipients were excluded from analysis. Data were analyzed from February 3, 2021, through February 26, 2022.

**Exposures:**

MOUD.

**Main Outcomes and Measures:**

Associations between cooccurring SUD diagnoses with treatment type were assessed with multivariable regression. The association of drug-related poisoning admissions with days covered with buprenorphine or naltrexone prescriptions vs days without prescriptions was assessed among MOUD initiators. Odds ratios from within-person fixed effects models were estimated as a function of MOUD and stratified by cooccurring SUDs.

**Results:**

Among 179 280 individuals with OUD (mean [SD] age, 33.2 [11.0] years; 90 196 [50.5%] men), 102 930 (57.4%) received psychosocial treatment without MOUD. Across 47 488 individuals with cooccurring SUDs, 33 449 (70.4%) did not receive MOUD, whereas across 131 792 individuals without cooccurring SUDs, 69 481 (52.7%) did not receive MOUD. Cooccurring SUD was associated with decreased odds of initiating buprenorphine (risk ratio [RR], 0.55 [95% CI, 0.54-0.56]) but increased odds of initiating naltrexone (extended release: RR, 1.12 [95% CI, 1.05-1.20]; oral: RR, 1.95 [95% CI, 1.86-2.03]). Among 12 485 individuals initiating MOUD who experienced at least 1 drug-related poisoning during insurance enrollment, buprenorphine treatment days were associated with decreased poisonings compared with days without MOUD for individuals with cooccurring SUD (odds ratio [OR], 0.56 [95% CI, 0.48-0.65]) and individuals without cooccurring SUD (OR, 0.57 [95% CI, 0.53-0.63]), with comparable associations observed for extended-release naltrexone. No protective association was observed for oral naltrexone.

**Conclusions and Relevance:**

These findings suggest that individuals with OUD and polysubstance use were less likely to initiate buprenorphine and naltrexone than individuals without polysubstance use. Among individuals initiating MOUD, polysubstance use was associated with decreased buprenorphine and increased naltrexone initiation, despite buprenorphine’s protective associations against drug-related poisoning.

## Key Points

**Question:**

What is the prevalence and comparative effectiveness associated with buprenorphine and naltrexone in individuals with opioid use disorder (OUD) and cooccurring substance use disorders (SUDs)?

**Findings:**

This comparative effectiveness study evaluated 179 280 individuals with OUD using insurance claims. Individuals with OUD and co-occurring SUDs were less likely to receive buprenorphine and more likely to receive extended-release naltrexone than peers without polysubstance use; buprenorphine and extended-release naltrexone were comparable in their protective associations with drug-related poisonings in both populations.

**Meaning:**

These findings suggest that individuals with cooccurring SUDs were less likely to receive buprenorphine despite buprenorphine’s association with protecting against overdose in this population.

## Teaser

This comparative effectiveness study evaluates distribution of buprenorphine and naltrexone initiation among individuals with opioid use disorder with or without cooccurring substance use disorder.

## Introduction

The opioid overdose crisis in the US has evolved to become an epidemic of polysubstance use, with opioids more commonly misused with alcohol, stimulants, and sedatives than alone.^1,2^ Despite a higher likelihood of treatment discontinuation and overdose,^3^ polysubstance use in individuals with opioid use disorder (OUD) is understudied and undertreated.^3,4^

To date, patterns of buprenorphine and naltrexone use among individuals with OUD and cooccurring substance use disorders (SUD) has not been well-characterized in national samples in the US. While medication for OUD (MOUD) receipt is posited to be lower in people with polysubstance use, the comparative effectiveness of different MOUDs has not been thoroughly investigated in such individuals.^5,6^ In the case of buprenorphine, individuals with cooccurring SUDs have historically been deemed poor candidates for buprenorphine treatment, although recent efforts have challenged such an approach to treatment.^7^ In the case of naltrexone, opioid antagonists have garnered US Food and Drug Administration indications for both OUD and alcohol use disorder (AUD); however, the effectiveness of naltrexone in treating OUD in individuals with cooccurring SUD is not well studied, including potential protection against other drug-related adverse events. While studies have suggested that opioid antagonists, such as naltrexone, lessen reinforcing effects of multiple addictive substances, including from alcohol,^8^ opioids,^9^ stimulants,^10,11^ and sedatives,^12^ high treatment discontinuation rates associated with naltrexone in studies of individuals with OUD^6^ have raised questions about whether buprenorphine may hold similar promise in treating OUD in people with cooccurring SUD. Research examining the comparative effectiveness of buprenorphine and naltrexone in individuals with polysubstance use is thus needed.

Using a large national cohort of commercial insurance and Medicaid enrollees, we evaluated the association between polysubstance use with buprenorphine and naltrexone use in individuals with a primary diagnosis of OUD. Among individuals receiving buprenorphine or naltrexone, we examined the association of buprenorphine and naltrexone use with nonfatal drug-related poisonings in individuals with OUD and polysubstance use.

## Methods

This comparative effectiveness study was deemed exempt from human participants review and informed consent by the Washington University institutional review board because no identifiable private data were used. This study is reported following the Strengthening the Reporting of Observational Studies in Epidemiology (STROBE) reporting guideline and the Reporting of Studies Conducted Using Observational Routinely Collected Health Data Statement for Pharmacoepidemiology (RECORD-PE) reporting guideline.

### Study Setting, Participants, and Observation Window

**Figure 1.**
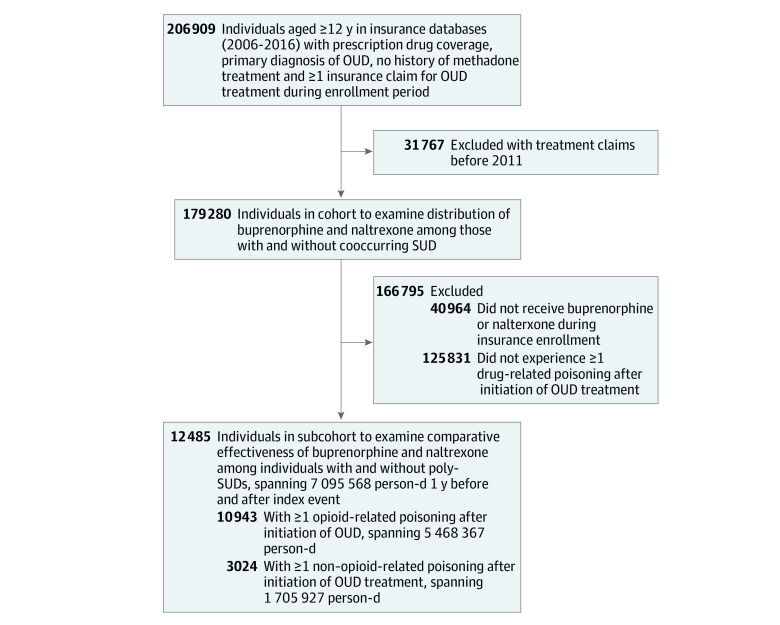
Flowchart of Derivation of Analytic Sample OUD indicates opioid use disorder; SUD, substance use disorder.

In this comparative effectiveness study, we used comprehensive insurance claims (ie, all paid services, including outpatient, inpatient, and pharmacy) from the IBM MarketScan Commercial and Multi-State Medicaid Databases from January 1, 2011, until December 31, 2016. Details on methods of data extraction have previously been described.^13^ Our analytic sample was derived from cohort of 206 909 individuals in the US aged 12 to 64 years with a primary OUD diagnosis and no previous methadone treatment, defined by *International Classification of Diseases, Ninth Revision* (*ICD-9*) and *International Statistical Classification of Diseases and Related Health Problems, Tenth Revision* (*ICD-10*) codes, who had at least 1 period of continuous insurance enrollment and corresponding treatment claim for 1 of the following: buprenorphine, extended-release (ER) naltrexone, oral naltrexone, or psychosocial treatment without MOUD. Each period of continuous insurance coverage ended when there was no claim for at least 45 days following the last dose of the most recent prescription assumed to be used. We subsequently limited our sample to 179 280 individuals who had a primary diagnosis of OUD and restricted the years of observation to 2011 to 2016, concomitant with the introduction of ER naltrexone for OUD in 2011 (Figure 1). We conducted retrospective analyses on this sample to evaluate the association between baseline cooccurring SUD, either concurrent with or in the 6-month preceding OUD treatment initiation, and subsequent initiation of buprenorphine or naltrexone during the individual’s first treatment episode. Individuals with history of methadone treatment were not included, as methadone treatment programs are characterized by close patient supervision and frequent drug testing^14^ that may confound observed associations between medication treatment and outcomes. The diagnosis of OUD was based on *ICD-9* and *ICD-10* codes (eTable 1 in the Supplement).

To examine the comparative effectiveness of MOUD using within-person fixed effects (case-crossover) models, we derived a subcohort of individuals from the analysis who experienced any drug-related poisoning, defined as emergency department (ED) visit or inpatient hospitalization and received any MOUD. This excluded 40 964 individuals who never received MOUD (naltrexone or buprenorphine) during insurance coverage. We excluded 125 831 individuals who did not experience a drug-related poisoning (outcome of interest) during coverage. We created an observation window (period during which an individual was assessed for drug exposure) limited to 1 year before and after a person’s first (index) event to reduce heterogeneity in observation time, culminating in 12 485 individuals encompassing 7 095 568 person-days.

### Variables

For the analysis of polysubstance use and its association with MOUD initiation, the primary outcome was OUD treatment type, operationalized as mutually exclusive categories of medication type (ie, buprenorphine, oral naltrexone, or ER naltrexone) or psychosocial treatment without MOUD (reference group). The exposure variable was presence of a cooccurring SUD in the 6-month preceding treatment, spanning alcohol, stimulant (cocaine and/or amphetamines), and/or sedative use disorder, as well as individuals without recent SUD diagnoses.

For the analysis of whether the protective associations of buprenorphine or naltrexone varied based on presence of cooccurring SUD, the primary outcome variable was hospitalizations or ED visits for any nonfatal drug-related poisoning (eTable 1 in the Supplement), derived from the US Center for Disease Control and Prevention listing of *ICD-9* and *ICD-10* codes pertaining to poisoning and pain.^15^ Secondary outcomes included opioid and nonopioid poisonings; drug-related poisonings that did not include opioid-specific codes were classified as nonopioid related poisonings.^15^ The exposure variables were medication use (buprenorphine or naltrexone [oral or ER]), coded as time-varying at the day level. Individuals were permitted to have multiple outcome events as long as they fell within 1 year after the index event. To mitigate misclassification of acute events, we limited place-of-service codes to hospital settings, EDs, ambulances, and inpatient psychiatry facilities, as opposed to long-term care, office-based, and residential facilities. We coded case-days as “1,” encompassing the index event and subsequent events, whereas control-days were coded as “0,” encompassing remaining days in the observation window. Assuming prescription fills denoted consumption of medication, we imputed medication treatment over time defined by presence or absence of filled prescriptions; national drug codes and procedure codes for buprenorphine and naltrexone have been detailed elsewhere.^13^ We coded medication days as “1” and nonmedication days as “0” (reference group). To assess polysubstance use, we used *ICD-9* and *ICD-10* codes during encounters to identify cooccurring SUDs at the time of OUD pharmacologic treatment initiation or during the 6 months preceding treatment. We subsequently stratified between participants with OUD and cooccurring SUD, as well as individuals without recent SUD diagnoses. Finally, we extracted data on age, sex, self-reported race and ethnicity, cooccurring psychiatric diagnoses (ie, mood, anxiety, psychotic, and personality disorders), Charlson comorbidity index, and insurance status for purposes of sample description.

### Statistical Analysis

We first conducted an analysis evaluating the association of buprenorphine or naltrexone initiation during treatment in association with baseline cooccurring SUD diagnoses (assessed as 6 months prior to, and inclusive of, OUD treatment initiation). Descriptive statistics evaluating the prevalence of buprenorphine and naltrexone use were conducted, stratifying for cooccurring SUD diagnoses for all individuals with a principal diagnosis of OUD, which is the first diagnosis recorded in an inpatient or outpatient record (Table 1; eTable 3 in the Supplement) and for the subcohort of medication recipients who had drug-related poisonings (eTable 2 and eTable 4 in the Supplement). The association of cooccurring SUDs with MOUD initiation (buprenorphine, ER naltrexone, and oral naltrexone vs psychosocial treatment without MOUD) was estimated using log-binomial regression through generalized estimating equations with logarithmic link functions, controlling for age, Medicaid status, and sex; we modeled the association of any cooccurring SUD and specific SUDs (alcohol, stimulant, and sedative use disorders) separately.

**Table 1.** Characteristics of Treatment-Seeking Individuals With a Primary Diagnosis of OUD

Characteristic	No. (%)		
	All (N = 179 280)	Any cooccurring SUD^a^	
		Yes (n = 47 488)	No (n = 131 792 )
Type of OUD treatment during first treatment episode			
Psychosocial treatment without medication	102 930 (57.4)	33 449 (70.4)	69 481 (52.7)
Buprenorphine	67 292 (37.5)	9651 (20.3)	57 641 (43.7)
ER naltrexone	3091 (1.7)	1156 (2.4)	1935 (1.5)
Oral naltrexone	5967 (3.3)	3232 (6.8)	2735 (2.1)
Sex			
Men	90 196 (50.3)	24 968 (52.6)	65 228 (49.5)
Women	89 084 (49.7)	22 520 (47.4)	66 564 (50.5)
Insurance			
Private	84 136 (46.9)	20 961 (44.1)	63 175 (47.9)
Medicaid	95 144 (53.1)	26 527 (55.9)	68 617 (52.1)
Race and ethnicity^b^			
Black	6362 (7.1)	2271 (9.1)	4091 (6.3)
Hispanic	1054 (1.2)	326 (1.3)	728 (1.1)
White	75 012 (83.6)	20 239 (81.4)	54 773 (84.4)
Other	7318 (8.2)	2038 (8.2)	5280 (8.1)
Age, mean (SD), y	33.2 (11.0)	33.4 (11.5)	33.1 (10.8)
Nonopioid cooccurring substance use			
Alcohol use disorder	30 767 (17.2)	30 767 (64.8)	NA
Stimulant use disorder	26 125 (14.6)	26 125 (55.0)	NA
Sedative use disorder	16 913 (9.4)	16 913 (35.6)	NA
Mental health condition			
Mood disorder	73 255 (40.9)	26 572 (56.0)	46 683 (35.4)
Psychotic disorder	5730 (3.2)	3149 (6.6)	2581 (2.0)
Personality disorder	5105 (2.9)	2740 (5.8)	2365 (1.8)
Anxiety disorder	62 036 (34.6)	21 532 (45.3)	40 504 (30.7)
Charlson comorbidity index			
0	160 267 (89.4)	40 136 (84.5)	120 131 (91.2)
1	13 923 (7.8)	5197 (10.9)	8726 (6.6)
2	3144 (1.8)	1232 (2.6)	1912 (1.5)
≥3	1946 (1.1)	923 (1.9)	1023 (0.8)

Abbreviations: NA, not applicable; OUD, opioid use disorder; SUD, substance use disorder.

^a^ SUDs were considered cooccurring in the 6 months preceding or concurrent with OUD treatment initiation.

^b^ Race and ethnicity data were available only for claims from Medicaid databases. Other race or ethnicity included people who did not disclose or who fell into a category other than Black, Hispanic, or White.

Among the subcohort of individuals who received buprenorphine or naltrexone and had at least 1 drug-related poisoning, we conducted within-person longitudinal analyses to evaluate whether buprenorphine or naltrexone use was associated with decreased risk of such events, both among individuals with and without cooccurring SUDs. This analysis used a repeatable-event, case-crossover design previously described in detail.^16^ In brief, the case-crossover method characterizes each person who experiences the outcome event over time by the presence or absence of treatment with medications. Case periods included days when acute events occurred; control periods encompassed days when such events did not occur, with each individual used as their own control by comparing buprenorphine or naltrexone prescriptions (exposure variable) at the time of event with exposure during control periods (no event). As a within-person analysis, the case-crossover design implicitly controls time-invariant confounding, although time-varying confounding is still an issue. The repeatable-event approach partially mitigates this because time can be included as a covariate.^17^ The association between drug-related poisoning and medication (buprenorphine or naltrexone) treatment days was estimated using conditional logistic regression.

To evaluate robustness of findings, we estimated models that included statins (negative control not expected to influence drug-related poisoning risk) and benzodiazepines (which are common and known to interact with buprenorphine in contributing to increased drug-related poisonings^16^) as time-varying covariates. We conducted sensitivity analyses limiting analyses to individuals who had an insurance claim for an OUD diagnosis in the 6 months preceding or including treatment initiation, as well as analyses excluding individuals with cooccurring alcohol use disorder to elucidate associations of naltrexone specifically, given its dual indications for alcohol use disorder and OUD.

Analyses were conducted using SAS statistical software version 9.4 (SAS Institute). *P* values were 2-sided, and statistical significance was set at *P* = .05. Data were analyzed from February 3, 2021, through February 26, 2022.

## Results

### Sample and Treatment Characteristics

Analyses included 179 280 individuals seeking OUD treatment (mean [SD] age, 33.2 [11.0] years; 90 196 [50.3%] men), with 6362 Black individuals (7.1%), 1054 Hispanic individuals (1.2%), and 75 012 White individuals (83.6%), and 7318 individuals (8.2%) identified as other race or ethnicity, including people who declined to answer or fell into categories other than Black, Hispanic, or White (Table 1). There were 47 488 individuals (26.5%) with a cooccurring SUD during the baseline period. Of all individuals, 67 292 (37.5%) received buprenorphine during the first treatment episode, compared with 3091 individuals (1.7%) receiving ER naltrexone and 5967 individuals (3.3%) receiving oral naltrexone. Additionally, 102 930 individuals (57.4%) received no buprenorphine or naltrexone during the first treatment episode, of whom 40 964 individuals (39.8%) did not receive any buprenorphine or naltrexone during insurance enrollment. With regards to cooccurring mental health conditions overall, 62 036 individuals (34.6%) had a diagnosis for an anxiety order and 73 255 individuals (40.9%) had a diagnosis for a mood disorder; psychotic (5730 individuals [3.2%]) and personality (5105 individuals [2.9%]) disorders were less common. Fewer than 5% of individuals had a Charlson comorbidity index greater than 2. Table 1 depicts descriptive summaries of clinical and demographic characteristics among all individuals with a primary diagnosis of OUD.

Of 47 488 individuals with a cooccurring SUD, 30 767 participants (64.8%) had alcohol use disorder, 26 125 individuals (55.0%) had stimulant use disorder, and 16 913 individuals (35.6%) had sedative use disorder at the start of the first treatment episode (Table 1; eTable 2 in the Supplement). The subcohort used in the within-person (case-crossover) analysis of drug-related poisonings in association with medication treatment periods is described in eTable 2 and eTable 4 in the Supplement; of 12 485 individuals who experienced drug-related poisonings, 10 502 individuals (84.1%) had more than 1 event.

A total of 33 449 individuals (70.4%) with cooccurring SUD during baseline received treatment without MOUD during first treatment episode, compared with 69 481 individuals (52.7%) without cooccurring SUD (Table 1). Whereas 9651 individuals (20.3%) with cooccurring SUD received buprenorphine, 57 641 individuals (43.7%) without cooccurring SUD received buprenorphine. Conversely, whereas among those with cooccurring SUD, 1156 individuals (2.4%) received ER naltrexone and 3232 individuals (6.8%) received oral naltrexone, among those without cooccurring SUD, only 1935 individuals (1.5%) received ER naltrexone and 2735 individuals (2.1%) received oral naltrexone.

### Association of Polysubstance Use With MOUD Initiation

Multivariable log-binomial analyses of the association between cooccurring SUD and medication initiation during the first treatment episode while controlling for age, Medicaid status, and sex are shown in Table 2. The presence of any cooccurring SUD was associated with decreased likelihood of buprenorphine treatment at time of OUD treatment initiation (risk ratio [RR], 0.55 [95% CI, 0.54-0.56]), compared with increased likelihood of ER naltrexone (RR, 1.12 [95% CI, 1.05-1.20]) and oral naltrexone (RR, 1.95 [95% CI, 1.86-2.03]) treatment initiation. In model 2 assessing individual cooccurring SUDs, there was decreased likelihood of buprenorphine initiation with cooccurring alcohol use disorder (RR,  0.60 [95% CI, 0.59-0.61]), stimulant use disorder (RR, 0.66 [95% CI, 0.65-0.68]), and sedative use disorder (RR, 0.73 [95% CI, 0.72-0.75]). While cooccurring alcohol use disorder was associated with increased initiation of ER naltrexone (RR, 1.22, [95% CI, 1.13-1.31]) or oral naltrexone (RR, 2.11 [95% CI, 2.02-2.20]), we did not observe an association between stimulant or sedative use disorders and naltrexone initiation, apart from a decrease in ER naltrexone prescriptions in individuals with cooccurring sedative use disorder (RR, 0.90 [95% CI, 0.82-0.99]).

**Table 2.** Adjusted Odds of OUD Medication Receipt in Association with Cooccurring Substance Use Disorders Among All Treatment-Seeking Individuals With a Primary Diagnosis of Opioid Use Disorder

Parameter	Buprenorphine^a^		ER naltrexone^a^		Oral naltrexone ^a^	
	OR (95% CI)	*P* value	OR (95% CI)	*P* value	OR (95% CI)	*P* value
**Model 1**						
Any cooccurring SUD	0.55 (0.54-0.56)	<.001	1.12 (1.05-1.20)	<.001	1.95 (1.86-2.03)	<.001
**Model 2**						
Alcohol use disorder	0.60 (0.59-0.61)	<.001	1.22 (1.13-1.31)	<.001	2.11 (2.02-2.20)	<.001
Stimulant use disorder	0.66 (0.65-0.68)	<.001	0.93 (0.86-1.01)	.10	1.06 (1.00-1.11)	.05
Sedative use disorder	0.73 (0.72-0.75)	<.001	0.90 (0.82-0.99)	.002	0.95 (0.90-1.01)	.11

Abbreviations: ER, extended release; OR, odds ratio; SUD, substance use disorder.

^a^ For all analyses, ORs were calculated as the odds of receiving the medication compared with the reference group of no cooccurring use disorder.

### Polysubstance Use and Comparative Effectiveness Associated With MOUD

**Figure 2.**
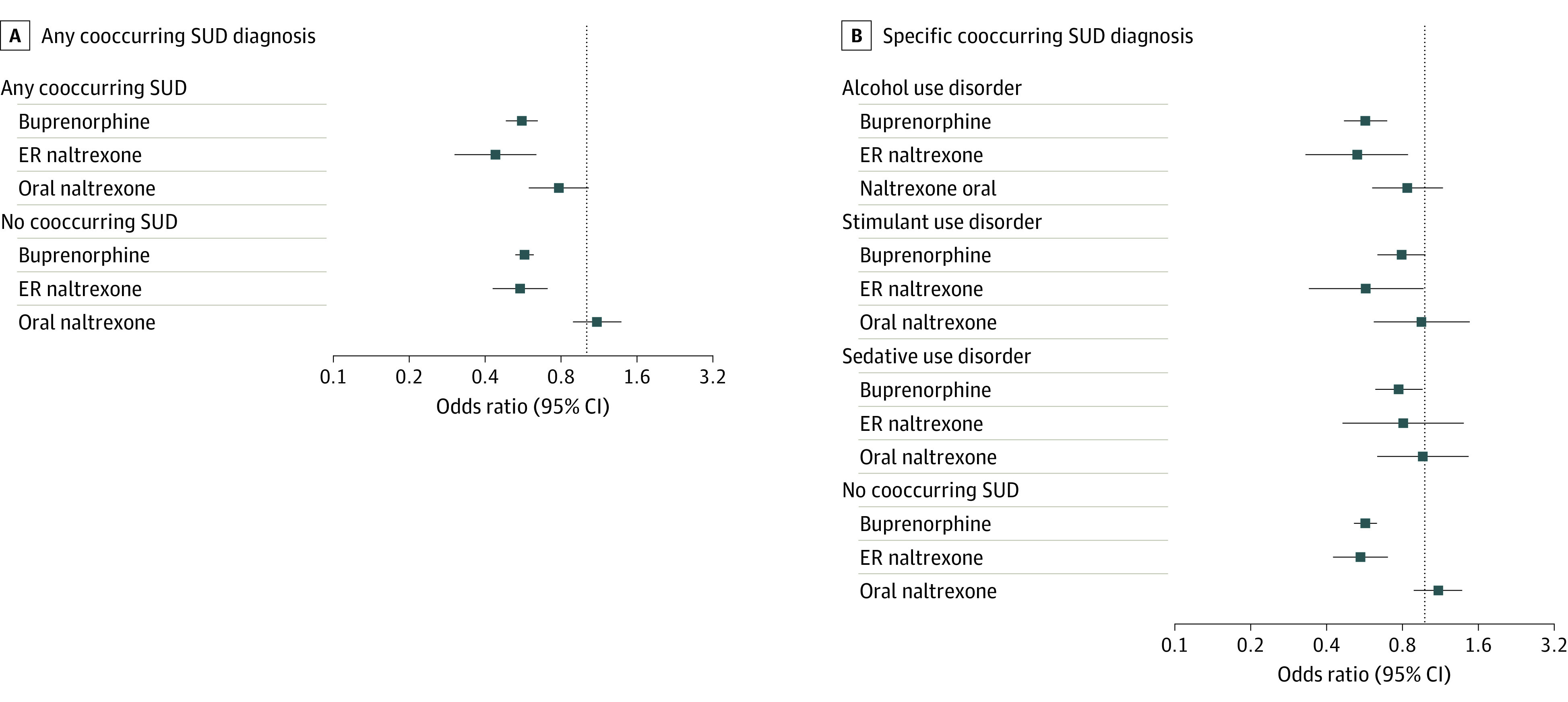
Odds of Acute Drug-Related Poisonings Associated With OUD Treatment Days Compared With Nontreatment Days, Stratified by Any Substance Use Disorder (SUD) Diagnosis During the 6 Months Preceding Treatment Initiation Odds ratios were calculated among 12 485 individuals encompassing 7 095 568 person-days. ER indicates extended release.

As depicted in Figure 2A, buprenorphine treatment days were associated with lower odds of hospitalization or ED visit for acute drug-related poisonings among individuals with cooccurring SUD (odds ratio [OR], 0.56 [95% CI, 0.48-0.65]) and those without cooccurring SUD (OR, 0.57 [95% CI, 0.53-0.63]). Comparable protective associations were observed in association with ER naltrexone treatment days for individuals with cooccurring SUD (OR, 0.44 [95% CI, 0.30-0.63]) but not for individuals without cooccurring SUD. No protective association was observed for oral naltrexone.

In secondary analyses stratifying by event subtype, buprenorphine was associated with decreased opioid-specific poisonings across individuals with cooccurring SUD (OR, 0.51 [95% CI, 0.43-0.61]) and without cooccurring SUD (OR, 0.49 [95% CI, 0.44-0.55]), with similar associations observed for ER naltrexone (eFigure in the Supplement). While oral naltrexone was associated with decreased opioid-specific admissions for individuals with cooccurring SUD (OR, 0.53 [95% CI, 0.36-0.77]), no association was observed among individuals without cooccurring SUD. Notably, while buprenorphine exhibited protective associations with decreased nonopioid poisonings among individuals with cooccurring SUD (OR, 0.64 [95% CI, 0.49-0.85]) and those without cooccurring SUD (OR, 0.79 [95% CI, 0.67-0.94]), no protective associations were observed for ER or oral naltrexone with nonopioid poisonings (eFigure in the Supplement).

In further secondary analyses, we evaluated the comparative effectiveness of MOUD with individual cooccurring SUDs, observing comparable protective associations with buprenorphine treatment days for individuals without cooccurring SUD (OR, 0.57 [95% CI, 0.53-0.63]) and those with alcohol use disorder (OR, 0.57 [95% CI, 0.47-0.69]), stimulant use disorder (OR, 0.78 [0.63-0.96]), and sedative use disorder (OR, 0.79 [95% CI, 0.64-0.98]) (Figure 2). While no association was observed between ER naltrexone and odds of drug-related poisoning for individuals with cooccurring sedative use disorder, comparable results were otherwise observed in all other comparisons (Figure 2). No protective association was observed for oral naltrexone.

These associations were sustained in sensitivity analyses controlling for benzodiazepines (positive control,) and statins (negative control) (eFigure in the Supplement), with univariate distributions of benzodiazepine and statin use (eTable 5 in the Supplement). The protective associations of buprenorphine and naltrexone were also sustained in analyses that limited events to those that occurred among individuals without a concurrent alcohol use disorder diagnosis (eFigure in the Supplement) and individuals with an active OUD diagnosis in the electronic medical record at the time of ED visit or hospitalization (eFigure in the Supplement).

## Discussion

In this comparative effectiveness study assessing individuals with OUD, more than 50% of individuals with OUD without cooccurring SUD and more 70% of individuals with cooccurring SUD did not receive evidence-based MOUD at time of treatment initiation. This indicates significant treatment gaps in a particularly high-risk population. While clinical studies have revealed similar efficacy of buprenorphine and naltrexone in treatment of OUD in per protocol analyses,^18^ our analysis found that the distribution of buprenorphine and naltrexone prescriptions in clinical practice varied based on the presence of a cooccurring SUD, such that individuals with cooccurring SUDs may be more likely to receive naltrexone and less likely to receive buprenorphine. Existing research shows polysubstance use is associated with lower likelihood of buprenorphine receipt,^3,13^ and although the American Society of Addiction Medicine has advised that cooccurring SUDs should not result in suspension of OUD treatment,^7^ polysubstance use remains associated with stigma, opioid agonist treatment underdosing,^19^ and exclusion from treatment.^1,20^ A 2020 analysis of US veterans reported lower likelihood of buprenorphine receipt associated with cooccurring SUDs.^3^ A 2021 analysis of US insurance claims found that opioid agonist therapy was underprescribed to individuals with OUD and alcohol use disorder.^13^ The level of abstinence historically required for patients to remain in buprenorphine treatment has been identified as a factor associated with low buprenorphine initiation and retention in treatment,^20^ potentially explaining low utilization rates among individuals with cooccurring SUDs.

In addition, we found consistent protective associations of both buprenorphine against drug-related poisonings across multiple types of cooccurring SUDs, with comparable associations observed for ER naltrexone. While naltrexone has been regarded as a promising treatment for individuals with OUD and polysubstance use, the effectiveness of naltrexone in clinical practice is limited by the requirement to complete detoxification prior to induction, elevated treatment discontinuation,^13^ and elevated overdose rates while receiving medication compared with buprenorphine.^5,6^ We found that oral naltrexone was not associated with significant protective associations against drug-related poisoning across most analyses; this is consistent with previous literature reporting high treatment discontinuation and overdose rates associated with oral naltrexone among individuals with OUD.^21^

### Limitations

This study has some limitations, including unmeasured confounding and measurement error, as prescription coverage and fills data do not always reflect actual consumption, as well as lower rates of naltrexone receipt compared with buprenorphine. Future research is needed to assess time using and not using medication (ie, treatment discontinuation risk). Although we adjusted for both calendar-time and restricted individuals to 2-year observation periods surrounding the index drug-related poisoning to reduce heterogeneity in observation time, unmeasured time-varying confounding is possible, such as engagement with the health care system, although we found no significant protective associations with statins (negative control). Bias resulting from control period selection as a function of event time cannot be ruled out, although our design benefited from bidirectional time sampling, with past results in this study cohort being robust to the choice of control period window.^16^ Our results are also limited by lack of race and ethnicity data in commercial insurance claims, limiting our ability to study the associations of structural racism in shaping outcomes in individuals with cooccurring SUD. These findings also predate the surge of synthetic opioid use in the late 2010s, further external validity.

Additionally, while recent studies^22^ have shown poor positive predictive values of *ICD* codes for OUD, our analysis was conducted on treatment-seeking individuals with a primary diagnosis of OUD who were likely to have a high severity of substance use and its concomitant impairment,^23^ rendering it more likely that our sample was correctly capturing individuals with active OUD. We also conducted sensitivity analyses that eliminated individuals with cooccurring alcohol use disorder and limited observations to those with active OUD claims, illustrating robustness of our main effects. Our study is also strengthened by use of repeatable-event case-crossover design, mitigating bias from time-invariant confounding, as each participant acted as his or her own control.

## Conclusions

The findings of this comparative effectiveness study provide insight into the high-risk and understudied population of individuals with OUD and cooccurring SUDs during a time when the opioid overdose epidemic is evolving into a polysubstance use crisis. We found that individuals with OUD and polysubstance use had a lower distribution of buprenorphine initiation even though buprenorphine demonstrated potentially more robust protective associations against acute drug-related events compared with ER naltrexone. Targeted efforts are needed to expand access to MOUD in individuals with polysubstance use.

## Notes

Supplement. **eMethods.** Description of Conditional Logistic Regression **eTable 1.** Diagnosis Codes for OUD and Drug-Related Poisonings, Procedural Codes for OUD Treatment Services, and NDC Codes Used for Identification of OUD Pharmacy Claims **eTable 2.** Treatment Characteristics at the Individual Participant Level for All Individuals with a Primary Diagnosis of OUD and Acute Drug-Related Events During 1 Year Before and After Index Acute Event **eTable 3.** Treatment Characteristics at the Individual Participant Level for All Individuals with OUD **eTable 4.** Treatment Characteristics at the Individual Participant Level for Persons with OUD and Acute Drug-Related Poisonings, During 1 Year Before and After Index Event **eTable 5.** Distribution of Benzodiazepine and Statin Use During 1 Year Before and After Index Acute Drug-Related Poisoning **eFigure.** Sensitivity Analyses of Drug-Related Poisoning Associated with Opioid Use Disorder Treatment Days Compared with Nontreatment Days
