# Supplementary material for: Comparative Effectiveness Associated With Buprenorphine and Naltrexone in Opioid Use Disorder and Cooccurring Polysubstance Use
Source: JAMA Netw Open. 2022 May 10;5(5):e2211363. doi: 10.1001/jamanetworkopen.2022.11363 (PMC9092203; doi:10.1001/jamanetworkopen.2022.11363)
Supplement: Supplement. — eMethods. Description of Conditional Logistic Regression eTable 1. Diagnosis Codes for OUD and Drug-Related Poisonings, Procedural Codes for OUD Treatment Services, and NDC Codes Used for Identification of OUD Pharmacy Claims eTable 2. Treatment Characteristics at the Individual Participant Level for All Individuals with a Primary Diagnosis of OUD and Acute Drug-Related Events During 1 Year Before and After Index Acute Event eTable 3. Treatment Characteristics at the Individual Participant Level for All Individuals with OUD eTable 4. Treatment Characteristics at the Individual Participant Level for Persons with OUD and Acute Drug-Related Poisonings, During 1 Year Before and After Index Event eTable 5. Distribution of Benzodiazepine and Statin Use During 1 Year Before and After Index Acute Drug-Related Poisoning eFigure. Sensitivity Analyses of Drug-Related Poisoning Associated with Opioid Use Disorder Treatment Days Compared with Nontreatment Days [file jamanetwopen-e2211363-s001.pdf]

Supplemental Online Content

Xu KY, Mintz CM, Presnall N, Bierut LJ, Grucza RA. Comparative effectiveness associated with buprenorphine and naltrexone in opioid use disorder and cooccurring polysubstance use. *JAMA Netw Open*. 2022;5(5):e2211363. doi:10.1001/jamanetworkopen.2022.11363

eMethods. Description of Conditional Logistic Regression

eTable 1. Diagnosis Codes for OUD and Drug-Related Poisonings, Procedural Codes for OUD Treatment Services, and NDC Codes Used for Identification of OUD Pharmacy Claims

eTable 2. Treatment Characteristics at the Individual Participant Level for All Individuals with a Primary Diagnosis of OUD and Acute Drug-Related Events During 1 Year Before and After Index Acute Event

eTable 3. Treatment Characteristics at the Individual Participant Level for All Individuals with OUD

eTable 4. Treatment Characteristics at the Individual Participant Level for Persons with OUD and Acute Drug-Related Poisonings, During 1 Year Before and After Index Event

eTable 5. Distribution of Benzodiazepine and Statin Use During 1 Year Before and After Index Acute Drug-Related Poisoning

eFigure. Sensitivity Analyses of Drug-Related Poisoning Associated with Opioid Use Disorder Treatment Days Compared with Nontreatment Days

This supplemental material has been provided by the authors to give readers additional information about their work.

© 2022 Xu KY et al. *JAMA Network Open.*

eMethods. Description of Conditional Logistic Regression

DEFINITIONS OF MEDICATION USAGE

Prescription use data was obtained from national drug codes in the MarketScan outpatient drug files. For each participant, we identified the first claim for medication fills, assuming that the prescription was filled on the date after the script was received by the pharmacy and consumed until the last day supplied. Furthermore, it was assumed that naltrexone extended-release was active for 28 days from date of injection. Persons who underwent detoxification and subsequently initiated on naltrexone were classified as having received naltrexone ER or naltrexone PO as opposed to no OUD medication. After individuals stopped taking medications, they were followed until the end of the observation window, spanning a maximum of 2 years. We did not delineate a minimum number of days' supply in order to meet a definition for usage. However, we required individuals to have medications filled subsequent to a primary OUD diagnosis within the 6 months from the beginning of treatment.

CONDITIONAL LOGISTIC REGRESSION

We estimated the association between drug-related poisonings and medication (buprenorphine or naltrexone) treatment days using conditional logistic regression among 12,485 individuals with OUD who received OUD medication and had at least one acute drug-related poisoning or injury during insurance coverage. We estimated the odds of an acute event associated with each day of medication fills compared to a day of non-treatment. Such an approach is analogous to a stratified Cox-regression with repeatable events (Xu et al., 2021). Because our analysis used a repeatable-event approach, we incorporated calendar-time as a covariate, mitigating confounding by secular time that can occur in single-event case-crossover studies (Allison 2006; Allison & Christakis 2006). The treatment effects among individuals with specific SUDs were estimated by coding the product of SUD diagnoses and a binary indicator for whether or not medication was present on a given day. Secular time trends were controlled using restricted cubic splines, using calendar-time as a covariate.

To assess the robustness of our findings, multivariable models were estimated that controlled for statins and benzodiazepines.

- Statins were selected as a negative control because they are medications that are not expected to have any relationship to substance use disorder-related acute admissions but may reflect engagement with healthcare. We also included statins (a medication intended to be taken persistently over time) to assess for persistent user bias, which can arise in situations when most of individuals with variance on the exposure variable change from non-medication use to persistent medication use. .
- Benzodiazepines were selected as a positive control, as they are commonly used by patients with OUD and are known to interact with buprenorphine in contributing to increased drug-related poisonings (Xu et al., 2021).

We applied two-sided P-values with a 5% significance level. Analyses were conducted from April 15 to March 1, 2022 using SAS version 9.4.

References:

- Allison P. Fixed Effects Regression Methods in SAS. Paper presented at: SUGI 31 Proceedings 2006; San Francisco, CA.
- Allison P, Christakis N.A. Fixed-effects methods for the analysis of nonrepeated events. *Sociological Methodology.* 2006;36:155-172.
- Xu KY, Borodovsky JT, Presnall N, et al. Association Between Benzodiazepine or Z-Drug Prescriptions and Drug- Related Poisonings Among Patients Receiving Buprenorphine Maintenance: A Case-Crossover Analysis. *Am J Psychiatry.* 2021:appiajp202020081174.

© 2022 Xu KY et al. *JAMA Network Open.*

eTable 1. Diagnosis Codes for OUD and Drug-Related Poisonings, Procedural Codes for OUD Treatment Services, and NDC Codes Used for Identification of OUD Pharmacy Claims

eTable 1A: Diagnosis Codes for Drug-Related Poisoning Events

Diagnosis	ICD-9 and ICD-10-CM diagnosis codes
All Drug-Related Poisoning	965-971, T39-T44, X40-X44, X60-X64, X85, Y10-Y14, E850-E855, E950, E962, E980
Opioid-Related Poisoning	T40, 965, X42, X62, Y12, E850
Non-Opioid Related Poisoning	964-971, T39, T41-T44, X40-T41, T43-X44, X60-X61, X63-X64, X85, Y10-Y11, Y13-Y14, E851-E855, E950, E962, E980

eTable 1B: Procedural Codes for OUD Treatment Services, where **a** refers to methadone administration. **b** refers to extended-release naltrexone administration**. c-e** refers to detoxification services. Services without footnotes denote psychosocial treatment.

Coding System	Procedural Code
HCPCS System	H0001-H0050, H1000-H1005, H1010-H1011, H2000-H2001, H2010- H2011-H2019, H2020[a], H2021-H2037, J2315[b], T1002, T1006-T1007, T1012, T1016, T2048
CPT Codes	90791, 90832, 90834, 90837, 90839- 90840, 90845-90847, 90853 , 90801-90802, 90804, 90806, 90808, 90810, 90812, 90814, 90816, 90818, 90821, 90823, 90826, 90828 ,90857
International Statistical Classification of Diseases	9464, 9465[c] ,9466-9467, 9468[d], 9469, HZ2ZZZZ[e], HZ30ZZZ- HZ39ZZZ, HZ3BZZZ , HZ40ZZZ-HZ49ZZZ, HZ4BZZZ

eTable 1C: National Drug Codes Used for Identification of OUD Pharmacy Claims

Medication	NDC Codes
Buprenorphine	00054017613, 00054017713, 00054018813, 00054018913, 00093537856, 00093537956, 00093572056, 00093572156, 00228315303, 00228315403, 00228315473, 00228315503, 00228315573, 00228315603, 00378092393, 00378092493, 00406192303, 00406192403, 00490005130, 12496120201, 12496120203, 12496120401, 12496120403, 12496120801, 12496120803, 12496121201, 12496121203, 12496127802, 12496128302, 12496130602, 12496131002, 16590066630, 16590066730, 23490927003, 35356000430, 38779088800, 38779088801, 38779088803, 38779088809, 42291017430, 42291017530, 43063018430, 49452129203, 49452825301, 49452825302, 49452825303, 49452825304, 49999039515, 49999063830, 49999063930, 50383028793, 50383029493, 50383092493, 50383093093, 51927101200, 52959030430, 52959074930, 54123011430, 54123091430, 54123092930, 54123095730, 54123098630, 54569573900, 54569573901, 54569573902, 54569639900, 54868570700, 54868570701, 54868575000, 55700014730, 59385001201, 59385001230, 59385001401, 59385001430, 59385001601, 59385001630, 60429058630, 60429058730, 62991158301, 62991158302, 62991158303, 62991158304, 63275992202, 63370090506, 63370090509, 63370090510, 63370090515, 63874108503, 63874117303, 65162041503, 65162041603, 68071151003, 68308020830
Naltrexone PO	00056001130, 00056001170, 00185003901, 00185003930, 00406117001, 00406117003, 00555090201, 00555090202, 16729008101, 16729008110, 38779088703, 38779088704, 38779088705, 38779088706, 42291063230, 47335032683, 47335032688, 49452483501, 49452483502, 51224020630, 51224020650, 51285027501, 51285027502, 51552073701, 51552073702, 51927354800, 51927360200, 51927437700, 52152010502, 52152010530, 52372075102, 62991124301, 62991124302, 62991124303, 62991124304, 63370015810, 63370015815, 63370015825, 63370015835, 68084029121, 68094085362
Naltrexone ER	63459030042, 65757030001

© 2022 Xu KY et al. *JAMA Network Open.*

eTable 2. Treatment Characteristics at the Individual Participant Level for All Individuals with a Primary Diagnosis of OUD and Acute Drug-Related Events During 1 Year Before and After Index Acute Event

	All Individuals with OUD and Acute Drug-Related Poisoning, n=12,485	Any SUD in the 6 Months Preceding or Concurrent with OUD Treatment Initiation	
		Yes<br>n= 3,537 (28.3%)	No<br>n= 8,948 (71.7%)
OUD Treatment During Insurance Enrollment			
No OUD Meds	0	0	0
Buprenorphine	9,967 (79.8%)	2,433 (68.8%)	7,534 (84.2%)
Naltrexone ER	1,482 (11.9%)	542 (15.3%)	940 (10.5%)
Naltrexone PO	2,229 (17.9%)	980 (27.7%)	1,249 (14.0%)
OUD Treatment During 1 Year Before and After Index Acute Event			
No OUD Meds	7,376 (59.1%)	2,077 (58.7%)	5,299 (59.2%)
Buprenorphine	4,105 (32.9%)	1,060 (30.0%)	3,045 (34.0%)
Naltrexone ER	759 (6.1%)	262 (7.4%)	497 (5.6%)
Naltrexone PO	773 (6.2%)	305 (8.6%)	468 (5.2%)
Sex			
Male	6,509 (52.1%)	1,902 (53.8%)	4,607 (51.5%)
Female	5,976 (47.9%)	1,635 (46.2%)	4,341 (48.5%)
Insurance			
Private	7,733 (61.9%)	2,129 (60.2%)	5,604 (62.6%)
Medicaid	4,752 (38.1%)	1,408 (39.8%)	3,344 (37.4%)
Race (Among Medicaid only)			
Black	272 (6.2%)	96 (7.3%)	176 (5.7%)
Hispanic	55 (1.3%)	19 (1.5%)	36 (1.2%)
White	3,434 (77.9%)	1,011 (77.1%)	2,423 (78.2%)
Other	650 (14.7%)	186 (14.2%)	464 (15.0%)
Mean Age, years (sd)	30.1 (10.9)	30.6 (10.9)	29.9 (10.8)
Alcohol Use Disorder	2,412 (19.3%)	2,412 (68.2%)	0
Stimulant Use Disorder	1,854 (14.9%)	1,854 (52.4%)	0
Sedative Use Disorder	1,584 (12.7%)	1,584 (44.8%)	0
Mood Disorder	6,412 (51.4%)	2,382 (67.4%)	4,030 (45.0%)
Psychotic Disorder	556 (4.5%)	286 (8.1%)	270 (3.0%)
Personality Disorder	566 (4.5%)	308 (8.7%)	258 (2.9%)
Anxiety Disorder	5,193 (41.6%)	1,912 (54.1%)	3,281 (36.7%)
Charlson comorbidity index			
0	10,876 (87.1)	2,889 (81.7%)	7,987 (89.3%)
1	1,182 (9.5%)	466 (13.2%)	716 (8.0%)
2	258 (2.1%)	106 (3.0%)	152 (1.7%)
3 or greater	169 (1.4%)	76 (2.2%)	93 (1.05)

This table illustrates OUD treatment characteristics at the individual participant level among those with a history of acute drug-related poisoning during the study's observation window (1 year before and after index event). The prevalence of using specific medications of interest within the study's observation period is depicted, as well as demographic characteristics for participants.

1 Categories are not mutually exclusive

© 2022 Xu KY et al. *JAMA Network Open.*

eTable 3. Treatment Characteristics at the Individual Participant Level for All Individuals with OUD

		Specific SUD Diagnoses[1] in the 6 Months Preceding or Concurrent With OUD Treatment Initiation					
	All Individuals	Alcohol Use Disorder		Stimulant Use Disorder		Sedative Use Disorder	
		Yes n=30,767 (17.2%)	No n=148,513 (82.8%)	Yes n=26,125 (14.6%)	No n=153,155 (85.4%)	Yes n=16,913 (9.4%)	No n=162,367 (90.6%)
OUD Treatment During Insurance Enrollment							
No OUD Meds	102,930 (57.4%)	21,040 (68.4%)	81,890 (55.1%)	19,466 (74.5%)	83,464 (54.5%)	11,208 (66.3%)	91,722 (56.5%)
Buprenorphine	67,292 (37.5%)	6,180 (20.1%)	61,112 (41.2%)	4,930 (18.9%)	62,362 (40.7%)	4,319 (25.5%)	62,973 (38.8%)
Naltrexone ER	3,091 (1.7%)	841 (2.7%)	2,250 (1.5%)	528 (2.0%)	2,563 (1.7%)	384 (2.3%)	2,707 (1.7%)
Naltrexone PO	5,967 (3.3%)	2,706 (8.8%)	3,261 (2.2%)	1,201 (4.6%)	4,766 (3.1%)	1,002 (5.9%)	4,965 (3.1%)
Sex							
Male	90,196 (50.3%)	17,868 (58.1%)	72,328 (48.7%)	12,280 (47.0%)	77,916 (50.9%)	8,211 (48.6%)	81,985 (50.5%)
Female	89,084 (49.7%)	12,899 (41.9%)	76,185 (51.3%)	13,845 (53.0%)	75,239 (49.1%)	8,702 (51.5%)	80,382 (49.5%)
Insurance							
Private	84,136 (46.9%)	15,841 (51.5%)	68,295 (46.0%)	8,958 (34.3%)	75,178 (49.1%)	9,609 (56.8%)	74,527 (45.9%)
Medicaid	95,144 (53.1%)	14,926 (48.5%)	80,218 (54.0%)	17,167 (65.7%)	77,977 (50.9%)	7,304 (43.2%)	87,840 (54.1%)
Race (Among Medicaid only)							
Black	6,362 (7.1%)	1,413 (4.7%)	4,949 (3.4%)	1,501 (6.0%)	4,861 (3.3%)	329 (2.0%)	6,033 (3.8%)
Hispanic	1,054 (1.2%)	190 (0.6%)	864 (0.6%)	205 (0.8%)	849 (0.6%)	81 (0.5%)	973 (0.6%)
White	75,012 (83.6%)	11,099 (37.2%)	63,913 (44.4%)	13,121 (52.5%)	61,891 (41.6%)	5,972 (36.2%)	69,040 (43.9%)
Other	7,318 (8.2%)	1,323 (4.4%)	5,995 (4.2%)	1,187 (4.8%)	6,131 (4.1%)	512 (3.1%)	6,806 (4.3%)
Mean Age, years (sd)	33.2 (11.0)	34.4 (12.2)	33.0 (10.7)	32.1 (10.3)	33.4 (11.1)	32.1 (11.4)	33.3 (10.9)
Mood Disorder	73,255 (40.9%)	17,833 (58.0%)	55,422 (37.3%)	14,504 (55.5%)	58,751 (38.4%)	9,943 (58.8%)	63,312 (39.0%)
Psychotic Disorder	5,730 (3.2%)	2,027 (6.6%)	3,703 (2.5%)	2,088 (8.0%)	3,642 (2.4%)	979 (5.8%)	4,751 (2.9%)
Personality Disorder	5,105 (2.9%)	1,881 (6.1%)	3,224 (2.2%)	1,685 (6.5%)	3,420 (2.2%)	1,045 (6.2%)	4,060 (2.5%)
Anxiety Disorder	62,036 (34.6%)	14,369 (46.7%)	47,667 (32.1%)	11,773 (45.1%)	50,263 (32.8%)	9,318 (55.1%)	52,718 (32.5%)
Charlson comorbidity index							
0	160,267 (89.4%)	25,832 (84.0%)	134,435 (90.5%)	22,049 (84.4%)	138,218 (90.3%)	14,364 (84.9%)	145,903 (89.9%)
1	13,923 (7.8%)	3,425 (11.1%)	10,498 (7.1%)	2,930 (11.2%)	10,993 (7.2%)	1,871 (11.1%)	12,052 (7.4%)
2	3,144 (1.8%)	869 (2.8%)	2,275 (1.5%)	639 (2.5%)	2,505 (1.6%)	428 (2.5%)	2,716 (1.7%)
3 or greater	1,946 (1.1%)	641 (2.1%)	1,305 (0.9%)	507 (1.9%)	1,439 (0.9%)	250 (1.5%)	1,696 (1.0%)

1 Categories are not mutually exclusive

© 2022 Xu KY et al. *JAMA Network Open.*

eTable 4. Treatment Characteristics at the Individual Participant Level for Persons with OUD and Acute Drug-Related Poisonings, During 1 Year Before and After Index Event

		Specific SUD Diagnoses[1] in the 6 Months Preceding or Concurrent With OUD Treatment Initiation					
	All Individuals with OUD and Acute Drug-Related Poisoning, n=12,485	Alcohol Use Disorder		Stimulant Use Disorder		Sedative Use Disorder	
		Yes n=2,412 (19.3%)	No n=10,073 (80.7%)	Yes n=1,854 (14.9%)	No n=10,631 (85.2%)	Yes n=1,584 (12.7%)	No n=10,901 (87.3%)
OUD Treatment During Insurance Enrollment							
No OUD Meds	0	0	0	0	0	0	0
Buprenorphine	9,967 (79.8%)	1,604 (66.5%)	8,363 (83.0%)	1,292 (69.7%)	8,675 (81.6%)	1,172 (74.0%)	8,795 (80.7%)
Naltrexone ER	1,482 (11.9%)	359 (14.9%)	1,123 (11.2%)	306 (16.5%)	1,176 (11.1%)	207 (13.1%)	1,275 (11.7%)
Naltrexone PO	2,229 (17.9%)	747 (31.0%)	1,482 (14.7%)	457 (24.7%)	1,772 (16.7%)	399 (25.2%)	1,830 (16.8%)
OUD Treatment During 1 Year Before and After Index Acute Event							
No OUD Meds	7,376 (59.1%)	1,409 (58.4%)	5,967 (59.2%)	1,110 (59.9%)	6,266 (58.9%)	926 (58.5%)	6,450 (59.2%)
Buprenorphine	4,105 (32.9%)	713 (29.6%)	3,392 (33.7%)	545 (29.4%)	3,560 (33.5%)	498 (31.4%)	3,607 (33.1%)
Naltrexone ER	759 (6.1%)	173 (7.2%)	586 (5.8%)	148 (8.0%)	611 (5.8%)	109 (6.9%)	650 (6.0%)
Naltrexone PO	773 (6.2%)	235 (9.7%)	538 (5.3%)	140 (7.6%)	633 (6.0%)	129 (8.1%)	644 (5.9%)
Sex							
Male	6,509 (52.1%)	1,385 (57.4%)	5,124 (50.9%)	948 (51.1%)	5,561 (52.3%)	799 (50.4%)	5,710 (52.4%)
Female	5,976 (47.9%)	1,027 (42.6%)	4,949 (49.1%)	906 (48.9%)	5,070 (47.7%)	785 (49.6%)	5,191 (47.6%)
Insurance							
Private	7,733 (61.9%)	1,591 (66.0%)	6,142 (61.0%)	960 (51.8%)	6,773 (63.7%)	1,132 (71.5%)	6,601 (60.6%)
Medicaid	4,752 (38.1%)	821 (34.0%)	3,931 (39.0%)	894 (48.2%)	3,858 (36.3%)	452 (28.5%)	4,300 (39.5%)
Race (Among Medicaid only)							
Black	272 (6.2%)	52 (6.7%)	220 (6.1%)	74 (9.0%)	198 (5.5%)	15 (3.5%)	257 (6.5%)
Hispanic	55 (1.3%)	11 (1.4%)	44 (1.2%)	12 (1.5%)	43 (1.2%)	7 (1.6%)	48 (1.2%)
White	3,434 (77.9%)	593 (76.4%)	2,841 (78.2%)	620 (75.4%)	2,814 (78.4%)	346 (80.3%)	3,088 (77.6%)
Other	650 (14.7%)	120 (15.5%)	530 (14.6%)	116 (14.1%)	534 (14.9%)	63 (14.6%)	587 (14.8%)
Mean Age, years (sd)	30.1 (10.9)	31.3 (11.5)	29.9 (10.7)	29.7 (9.9)	30.2 (11.0)	29.6 (11.0)	30.2 (10.8)
Mood Disorder	6,412 (51.4%)	1,679 (69.6%)	4,733 (47.0%)	1,238 (66.8%)	5,174 (58.7%)	1,094 (69.1%)	5,318 (48.8%)
Psychotic Disorder	556 (4.5%)	201 (8.3%)	355 (3.5%)	171 (9.2%)	385 (3.6%)	128 (8.1%)	428 (3.9%)
Personality Disorder	566 (4.5%)	224 (9.3%)	342 (3.4%)	175 (9.4%)	391 (3.7%)	160 (10.1%)	406 (3.7%)
Anxiety Disorder	5,193 (41.6%)	1,348 (55.9%)	3,845 (38.2%)	1,026 (55.3%)	4,167 (39.2%)	1,001 (63.2%)	4,192 (38.5%)
Charlson comorbidity index							
0	10,876 (87.1)	1,969 (81.6%)	8,907 (88.4%)	1,482 (79.9%)	9,394 (88.4%)	1,319 (83.3%)	9,557 (87.7%)
1	1,182 (9.5%)	328 (13.6%)	854 (8.5%)	261 (14.1%)	921 (8.7%)	201 (12.7%)	981 (9.0%)
2	258 (2.1%)	68 (2.8%)	190 (1.9%)	64 (3.5%)	194 (1.8%)	37 (2.3%)	221 (2.0%)
3 or greater	169 (1.4%)	47 (2.0%)	122 (1.2%)	47 (2.5%)	122 (1.2%)	27 (1.7%)	142 (1.3%)

1 Categories are not mutually exclusive

© 2022 Xu KY et al. *JAMA Network Open.*

eTable 5. Distribution of Benzodiazepine and Statin Use During 1 Year Before and After Index Acute Drug-Related Poisoning

	All Individuals with OUD and Drug-Related Poisoning n=12,485 (100%)	Any SUD in the 6 Months Preceding or Concurrent with OUD Treatment Initiation	
		Yes n=3,537 (28.3%)	No n=8,948 (71.7%)
Benzodiazepine	1,930 (15.5%)	540 (15.3%)	1,390 (15.5%)
Statins	232 (1.9%)	81 (2.3%)	151 (1.7%)

	Alcohol Use Disorder		Stimulant Use Disorder		Sedative Use Disorder	
	Yes n=2,412 (19.3%)	No n=10,073 (80.7%)	Yes n=1,854 (14.9%)	No n=10,631 (85.2%)	Yes n=1,584 (12.7%)	No n=10,901 (87.3%)
Benzodiazepine	387 (16.0%)	1,543 (15.3%)	246 (13.3%)	1,684 (15.8%)	320 (20.2%)	1,610 (14.8%)
Statins	59 (2.5%)	173 (1.7%)	33 (1.8%)	199 (1.9%)	36 (2.3%)	196 (1.8%)

© 2022 Xu KY et al. *JAMA Network Open.*

eFigure 1: Sensitivity Analyses of Drug-Related Poisoning Associated with Opioid Use Disorder Treatment Days Compared with Nontreatment Days

1A

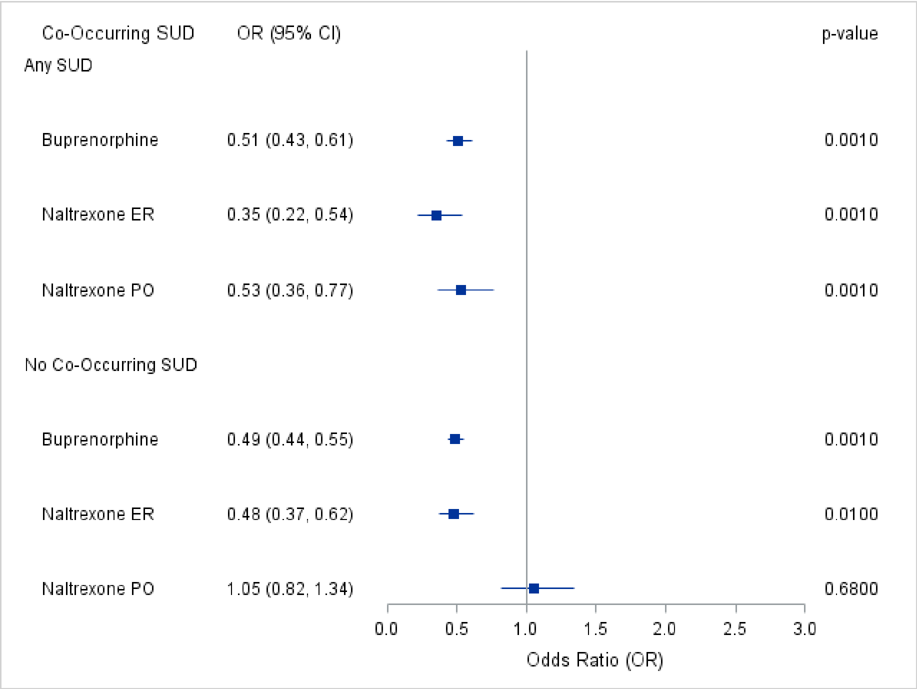

1B

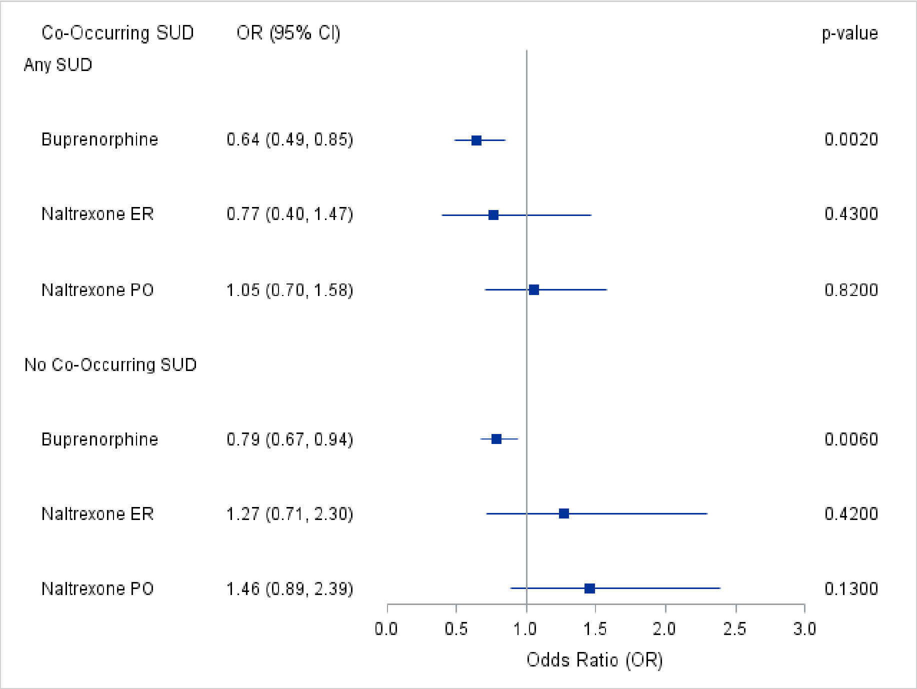

Odds of opioid-related poisonings associated with OUD treatment days stratified by any SUD diagnosis, n=10,943 individuals encompassing 5,468,367 person-days (eFigure 1A). Odds of non-opioid poisonings associated with OUD treatment days stratified by any SUD diagnosis, n=3,758 individuals encompassing 1,705,927 person-days (eFigure 1B).

© 2022 Xu KY et al. *JAMA Network Open.*

1C

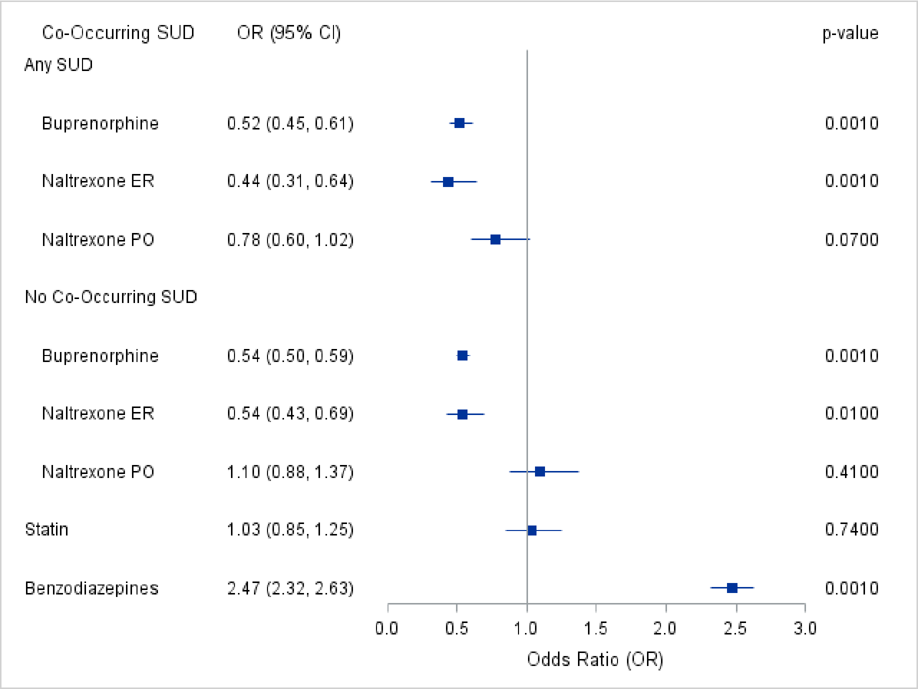

1D

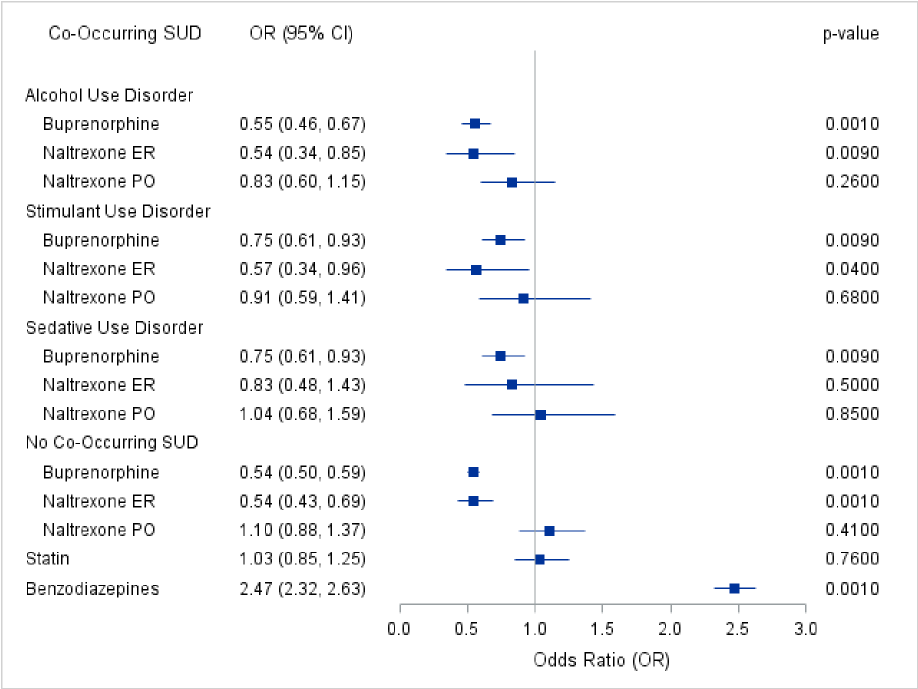

Odds of drug-related poisonings associated with opioid use disorder (OUD) treatment days compared with non-treatment days controlling for statins and benzodiazepines, stratified by any SUD diagnosis (eFigure 1C) during the 6 months preceding treatment initiation and stratified by specific SUD diagnoses (eFigure 1D), n=12,485 individuals encompassing 7,095,568 person-days

© 2022 Xu KY et al. *JAMA Network Open.*

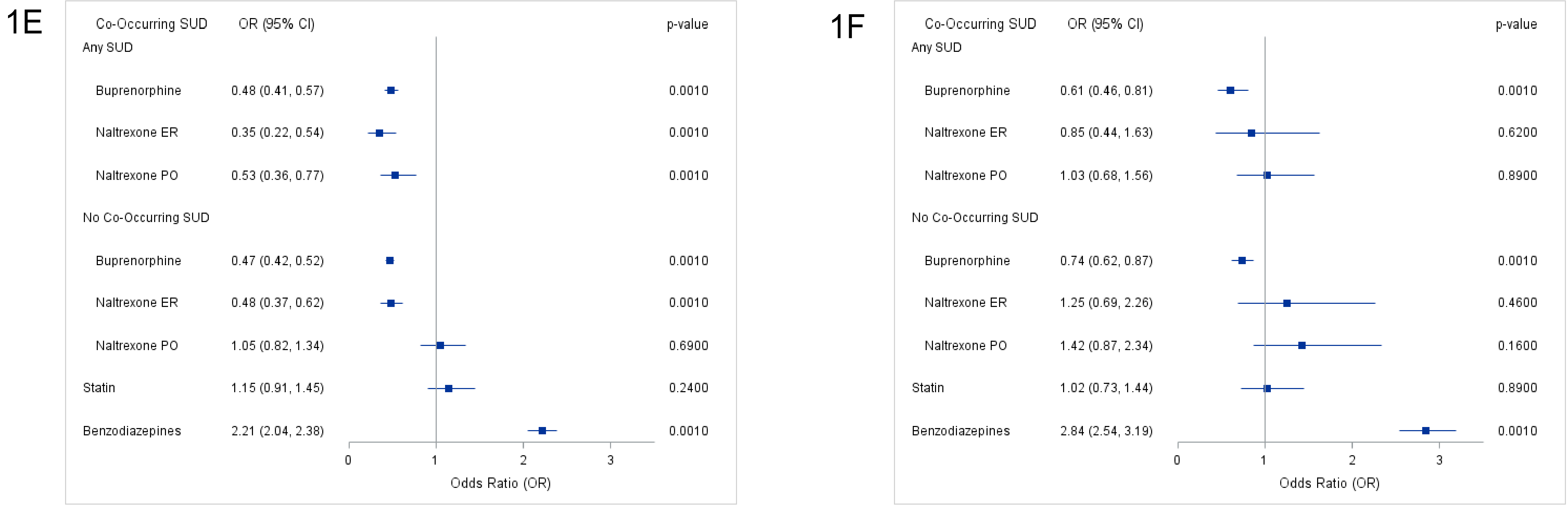

Odds of opioid-related poisonings associated with OUD treatment days stratified by any SUD diagnosis, n=10,943 individuals encompassing 5,468,367 person-days (eFigure 1E). Odds of non-opioid poisonings associated with OUD treatment days stratified by any SUD diagnosis, n=3,758 individuals encompassing 1,705,927 person-days (eFigure 1F).

© 2022 Xu KY et al. *JAMA Network Open.*

1G

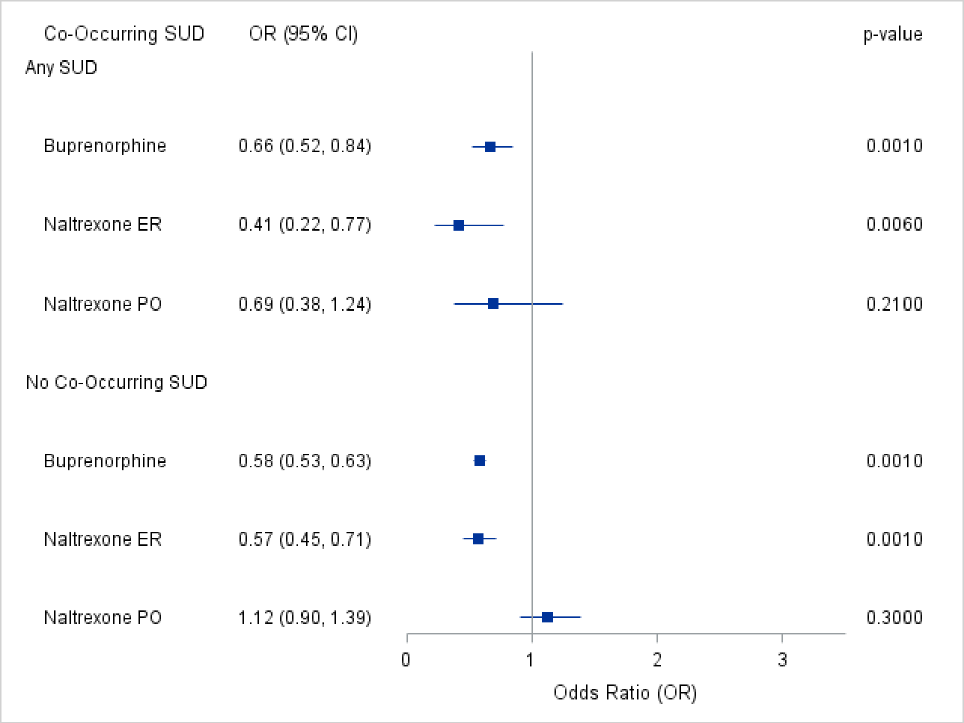

1H

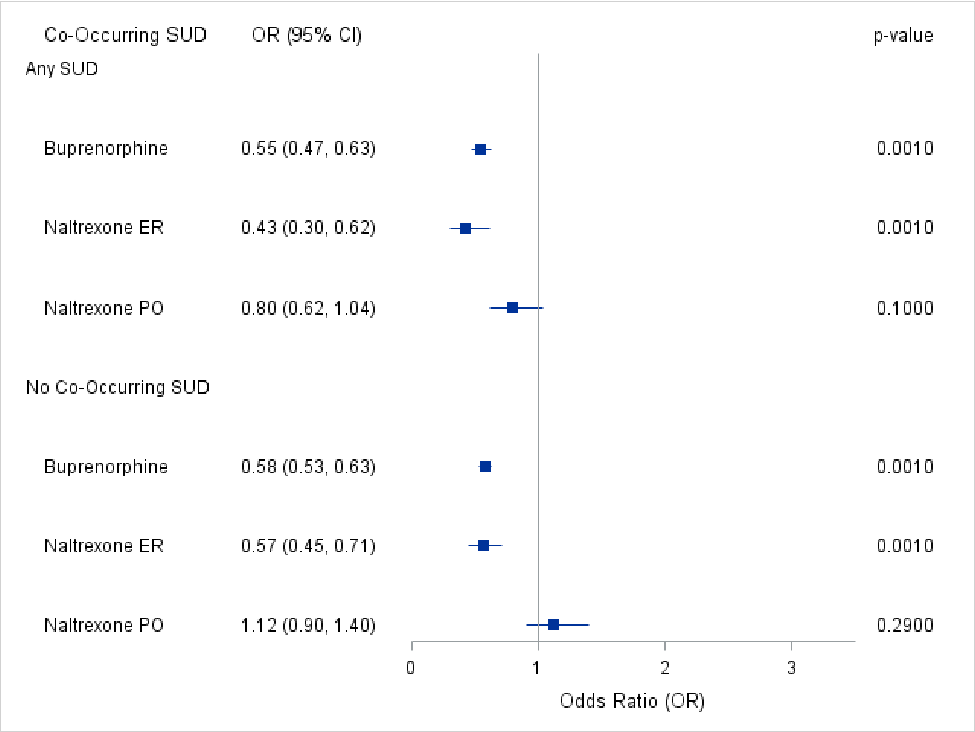

Odds of drug-related poisonings (excluding individuals with a co-occurring alcohol use disorder diagnosis) associated with OUD treatment days compared with non-treatment days, stratified by any SUD diagnosis, n= 10,327 individuals encompassing 5,870,958 person-days (eFigure 1G). Odds of drug-related poisonings (accompanied by an OUD diagnosis at the time of acute admission) associated with OUD treatment days compared with non-treatment days, stratified by any SUD diagnosis, n=12,782 individuals encompassing 7,273,390 person-days (eFigure 1H)

© 2022 Xu KY et al. *JAMA Network Open.*
